# Trajectory of depression occurrence before, during, and after dementia diagnosis: A population-based study

**DOI:** 10.1038/s41398-026-03817-w

**Published:** 2026-02-03

**Authors:** Wenzhe Yang, Weiwei Li, Sakura Sakakibara, Jiao Wang, Marc Guitart-Masip, Xiuying Qi, Abigail Dove, Weili Xu

**Affiliations:** 1 School of Public Health, Tianjin Medical University, Tianjin, China; 2 Aging Research Center, Department of Neurobiology, Care Sciences and Society, Karolinska Institutet, Stockholm, Sweden; 3 Center of Gerontology and Geriatrics, West China Hospital, Sichuan University, Chengdu, China; 4 National Clinical Research Center for Geriatrics, West China Hospital, Sichuan University, Chengdu, China; 5 Center for Psychiatry Research, Region Stockholm, Stockholm, Sweden; 6 Center for Cognitive and Computational Neuropsychiatry (CCNP), Karolinska Institutet, Stockholm, Sweden

**Keywords:** Depression, Scientific community

## Abstract

Depression and dementia commonly co-occur, yet little is known about depression trajectories across dementia stages. We aimed to map depression occurrence before, during, and after dementia diagnosis, and to identify factors associated with depression among individuals with dementia. This study included 10,051 participants from the Swedish Twin Registry. Participants with incident dementia (n = 2677) were matched with up to 3 controls (n = 7374) by birth year and sex. Depression and dementia diagnoses and their dates were ascertained based on medical records from the National Patient Registry. Conditional Poisson regression estimated incidence rate ratios for depression, while generalized estimating equations examined odds ratios for factors associated with depression. Compared with controls, depression risk among participants with dementia began to increase 6 years pre-diagnosis (incidence rate ratio [95% confidence interval] 2.32 [1.24–4.35]) and peaked during the year of dementia diagnosis (10.38 [7.33–14.69]). Depression risk remained elevated but gradually declined over the following 4 years (3.10 [1.67–5.77]). Female sex (odds ratio 2.21 [1.63–2.99]), smoking (1.58 [1.20–2.08]), heavy drinking (1.88 [1.10–3.21]), and stroke (1.94 [1.31–2.88]) were associated with higher odds of depression before dementia diagnosis, whereas being single (1.71 [1.10–2.37]) and having a history of cancer (1.35 [1.05–1.79]) were associated with post-diagnosis depression. Overall, these findings indicate that depression risk rises before, peaks at, and remains elevated after dementia diagnosis, with specific demographic (sex, marital status) and health-related factors (smoking, alcohol use, stroke, cancer) contributing to its occurrence among individuals with dementia.

## Introduction

Depression affects approximately 350 million people worldwide and is projected to become the leading global cause of disease burden by 2030 [1–3]. It is particularly prevalent among older adults, with up to 9.3% of individuals over the age of 60 experiencing depression [4]. Beyond seriously compromising social relationships and daily functioning, depression may also impair cognitive function and increase the risk of disability and suicide mortality among older adults [1, 5].

Both depression and cognitive impairment are common in late life and often co-occur [6–8]. While a recent meta-analysis of 19 longitudinal studies suggested that depression is associated with nearly a two-fold increased risk of dementia [6], other studies found that only late-life depression, but not earlier-life depression, is linked to dementia risk [9, 10]. However, traditional cohort study designs and time-to-event analyses are often limited by follow-up duration and changes in exposures over time. Given the complex relationship between the two disorders, it is challenging to determine whether depression occurring around the time of dementia diagnosis is a prodromal, independent, or secondary manifestation [7]. Anchoring the time scale of depression occurrence to a broader timeline of pre- and post-diagnosis of dementia, rather than relying solely on baseline assessment, can provide a clearer picture of their temporal relationship. Although some previous studies have examined the trajectory of depressive symptoms before dementia [11–13] or the prevalence of depression among individuals with dementia [14, 15], few have comprehensively mapped the risk of depression across the entire diagnostic timeline [16].

It has been suggested that depression risk in individuals with dementia differs from that in the general population [17]. The progression and worsening of cognitive impairment have a serious impact on an individual’s physical and mental health. These changes may further affect daily behavior and neurobiological regulation, thus potentially leading to differences in risk factors for post-dementia depression. However, comparative studies examining risk factors for depression before and after dementia diagnosis remain limited, with most existing research focusing solely on a single stage or on the general population [18–20].

In the present study, using data from the Swedish Twin Registry (STR), we aimed to map the trajectory of depression before, during, and after the diagnosis of dementia, and to explore which factors are associated with depression occurrence at different periods relative to dementia diagnosis.

## Methods

### Study population

**Fig. 1:**
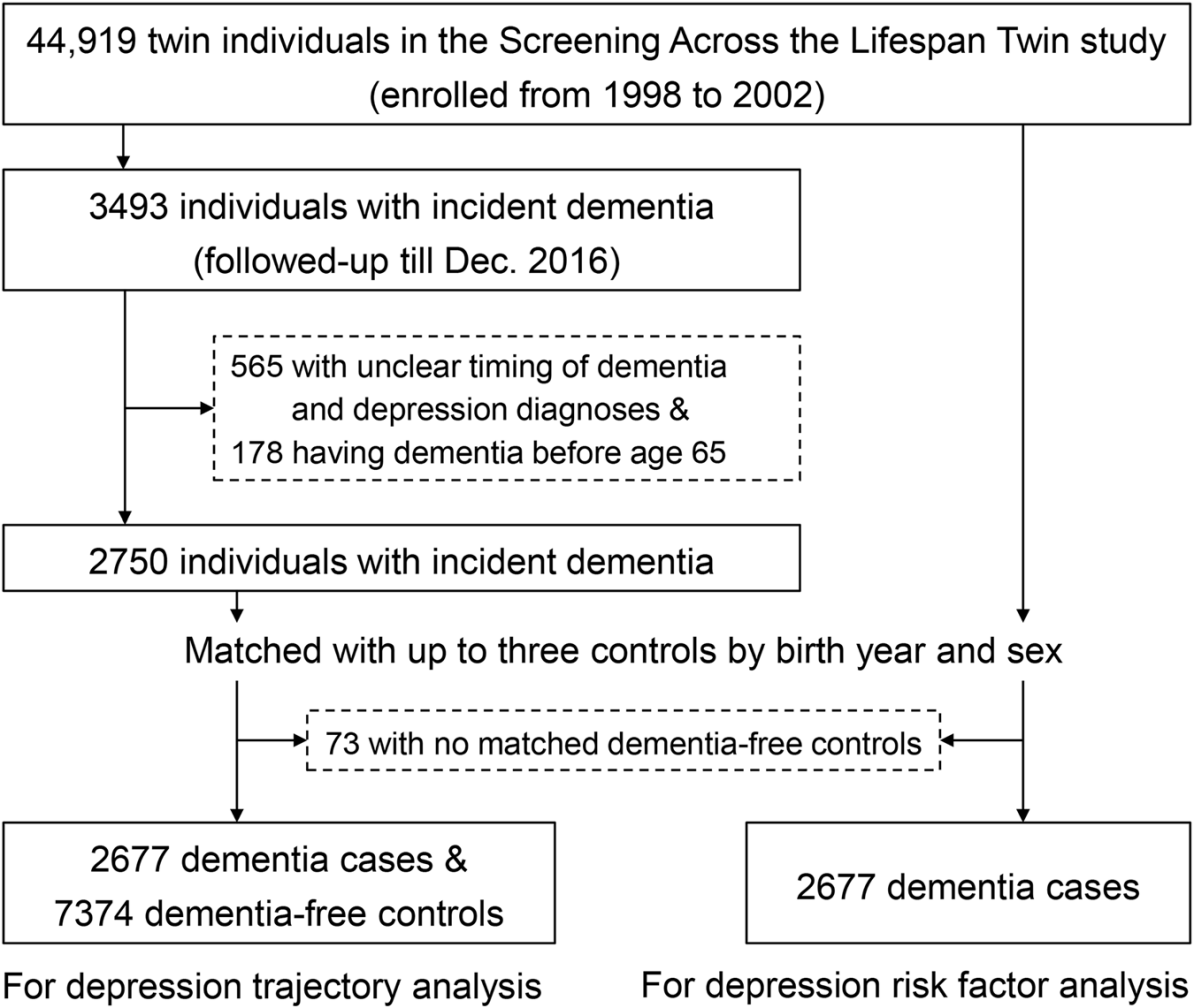
Flowchart of the study population.

Study participants were drawn from the STR [21]. Between 1998 and 2002, 44,919 twin individuals aged ≥40 were invited to participate in a computer-assisted telephone interview called the Screening Across the Lifespan Twin (SALT) survey. Changes in participants’ health status were subsequently monitored for up to 18 years (until December 2016) via linkage with medical records from the Swedish National Patient Register (NPR). First, among 3493 participants who developed dementia during the follow-up, we excluded 565 participants with unclear timing of dementia and depression diagnoses and 178 who developed dementia before age 65, leaving a total of 2750 participants with incident dementia. Next, these participants were matched with up to 3 dementia-free controls using propensity score matching based on year of birth and sex (73 participants with dementia could not be matched and were therefore excluded). A total of 10,051 individuals - including 2677 dementia cases and 7374 dementia-free controls - were included in the current study (Fig. 1).

All participants provided informed consent, and this study was approved by the Regional Ethics Board at Karolinska Institutet, Stockholm, Sweden.

### Data collection

Information on participants’ socio-demographic characteristics (sex, age, education level, and marital status), lifestyle behaviors (alcohol consumption, smoking history, and physical activity), zygosity status, height, weight, and medical history was collected through the SALT survey.

Education level was dichotomized as <8 years vs. ≥8 years based on the end of compulsory schooling for older Swedish cohorts at the time of their education [10]. Marital status was categorized as married/cohabitating vs. single (including divorced, widowed, and living alone). Smoking status was categorized as non-smokers vs. former/current smokers. Alcohol consumption was dichotomized as no/mild drinking vs. heavy drinking. Physical activity level was assessed through a survey question on annual exercise patterns and dichotomized as low (“almost never”, “much less than average”, and “less than average”) vs. high (“average”, “more than average”, “much more than average”, and “maximum”). Height and weight were collected based on self-report, and body mass index (BMI) was calculated as weight in kilograms (kg) divided by height in meters squared (m^2^).

Medical conditions, including hypertension, cancer, type 2 diabetes, heart disease, and stroke, were identified through self-report from the SALT, medication records from the Swedish Prescribed Drug Register, and/or diagnostic codes from the International Classification of Diseases (ICD) in the NPR (see Supplementary table 1 for the full list of ICD codes). The NPR covers all inpatient visits since 1987 and all specialized outpatient visits since 2001 [22]. ICD-8, −9, and −10 codes were used according to the corresponding periods of diagnostic coding in the NPR. Despite minor differences in coding criteria, all diagnoses were made by clinicians in specialized psychiatric and somatic care settings, ensuring high diagnostic validity and specificity [22, 23].

### Assessment of dementia

Dementia was identified based on diagnosis information extracted from the NPR. The ICD codes used for diagnoses of dementia included: ICD-8, 290; ICD-9, 290 A, 290B, 290E, 290 W, 290X, 331 A, 331 C; and ICD-10, F00, F01, F02, F03, F05, G30, G310, G318, G318A [24, 25]. The date of dementia occurrence was estimated according to the earliest recorded diagnosis date in the NPR.

### Ascertainment of depression

Depression was ascertained using data from the NPR, where diagnoses were made by psychiatrists in accordance with the Swedish adaptation of the ICD. The following ICD codes were used to identify depression cases: ICD-7, 314.99; ICD-8, 296.00, 298.00, 300.40, 300.41, 790.20; ICD-9, 296 C, 296D, 296 W, 298 A, 300E, 309 A, 309B, 311X; and ICD-10, F32, F33, F34.1, F41.2 [26, 27]. All depression records, together with their corresponding dates, were considered depression occurrences.

### Statistical analysis

Baseline characteristics of the participants by dementia status were compared using Chi-square (*χ*^2^) tests for categorical variables. In our analyses, the year of dementia diagnosis was set as the origin of the timescale to map the bidirectional trajectory of depression occurrence [28]. This means that year 0 corresponded to the year of dementia diagnosis for cases, and to the year at the corresponding age for controls. The interval from 10 to 1 years before dementia diagnosis (year -10 to year -1) was defined as the pre-diagnostic period, and the interval from 1 to 10 years after dementia diagnosis (year 1 to year 10) was defined as the post-diagnostic period.

In the primary analyses, conditional Poisson regression was used to estimate the annual incidence rates (IRs) of depression, standardized by age and sex, with 95% confidence intervals (CIs), from year −10 to year 10 in cases and matched controls. Incidence rate ratios (IRRs) with 95% CIs were also calculated. Models were adjusted for education level, marital status, physical activity level, hypertension, type 2 diabetes, heart disease, and stroke. These covariates were selected because their distributions differed significantly between individuals with and without dementia in our data. Age and sex were not included because they were accounted for through the matching design.

In the secondary analyses, generalized estimating equation (GEE) models were used to estimate odds ratios (ORs) and 95% CIs for the association between various sociodemographic, behavioral, and clinical factors and depression before and after dementia diagnosis. GEE models are considered more appropriate than logistic regression for capturing annual changes in depression occurrence in the pre- and post-diagnostic periods, and this analysis was conducted among the 2677 dementia cases. These potential factors included age, sex, marital status, education level, smoking status, alcohol consumption, physical activity level, BMI, hypertension, cancer, type 2 diabetes, heart disease, and stroke, which were selected based on their availability in the SALT survey and existing evidence linking them to depression in older adults [20, 26]. Age, hypertension, cancer, type 2 diabetes, heart disease, and stroke in the pre-diagnostic period were obtained at the time of the SALT survey and were reassessed 1 year after the diagnosis of dementia in the post-diagnostic period. Sex, education level, marital status, smoking status, alcohol consumption, physical activity, and BMI were only available from the SALT survey. All these factors were mutually adjusted in the GEE models.

Missing values for education (n = 704), marital status (n = 430), smoking status (n = 679), alcohol consumption (n = 705), physical activity (n = 2940), and BMI (n = 996) were handled through multiple imputation using the fully conditional specification method with 5 imputations. All analyses were conducted using SAS 9.4 (SAS Institute, Cary, NC, USA) and Stata SE 15.0 (StataCorp, College Station, TX, USA), with statistical significance defined as *P* < 0.05.

## Results

### Characteristics of the study population

In the study sample of 10,051 participants (including 2677 with incident dementia and 7374 dementia-free controls), 59.2% were women, and the mean (standard deviation) age at dementia diagnosis was 81.3 (6.5) years. Compared with dementia-free controls, those with incident dementia were more likely to be single, physically inactive, have a lower education level, and present with hypertension, type 2 diabetes, heart disease, and stroke. By contrast, BMI, smoking status, alcohol consumption, and cancer did not differ significantly between the two groups (Table 1).

**Table 1:** Basic characteristics of the study population by dementia status (n = 10,051).

Characteristics	Dementia-free (n = 7374)	Dementia (n = 2677)	*P*-value
Education level			<0.001
<8 years	3915 (53.1)	1540 (57.5)	
≥8 years	3459 (46.9)	1137 (42.5)	
Marital status			0.018
Married/cohabitating	4794 (65.0)	1672 (62.5)	
Single	2580 (35.0)	1005 (37.5)	
Zygosity			0.235
Monozygotic	1537 (20.9)	547 (20.4)	
Dizygotic	5141 (69.7)	1847 (69.0)	
Undetermined	696 (9.4)	283 (10.6)	
BMI, kg/m^2^			0.986
Underweight (<20)	396 (5.4)	147 (5.5)	
Normal weight (≥20 to <25)	3485 (47.3)	1268 (47.4)	
Overweight (≥25 to <30)	2909 (39.4)	1047 (39.1)	
Obese (≥30)	584 (7.9)	215 (8.0)	
Smoking			0.082
Non-smokers	4600 (62.4)	1619 (60.5)	
Current/former smokers	2774 (37.6)	1058 (39.5)	
Alcohol consumption			0.897
No/mild drinking	7075 (95.9)	2570 (96.0)	
Heavy drinking	299 (4.1)	107 (4.0)	
Physical activity level			0.022
Low	1213 (16.5)	492 (18.4)	
High	6161 (83.5)	2185 (81.6)	
Hypertension	415 (5.6)	221 (8.3)	<0.001
Cancer	937 (12.7)	324 (12.1)	0.419
Type 2 diabetes	523 (7.1)	272 (10.2)	<0.001
Heart diseases	914 (12.4)	447 (16.7)	<0.001
Stroke	369 (5.0)	231 (8.6)	<0.001

Data are presented as numbers (proportion, %).

### Trajectory of depression in relation to dementia diagnosis

In Poisson regression models, the IRs of depression among participants with and without dementia were similar (approximately 0.57–1.83 per 1000 person-years) during the 7 to 10 years preceding dementia diagnosis. Among participants with dementia, the incidence of depression began to increase 6 years before dementia diagnosis (IR 1.89 per 1000 person-years, 95% CI 1.01–2.76), reached a peak in the year of diagnosis (IR 13.06 per 1000 person-years, 95% CI 10.93–15.19), and then gradually decreased beginning 4 years after dementia diagnosis (approximately 2.94–5.37 per 1000 person-years) (Table 2).

**Table 2:** Age- and sex-standardized incidence rates (IRs) per 1000 person-years, incidence rate ratios (IRRs), and 95% confidence intervals (CIs) of depression before, during, and after dementia diagnosis (n = 10,051).

Time in relation to diagnosis (year)	Dementia-free (n = 7374)			Dementia (n = 2677)			IRR (95% CI) ^a^
	No. of participants	Depression cases (%)	IR (95% CI)	No. of participants	Depression cases (%)	IR (95% CI)	
−10	4886	12 (0.25)	0.57 (0.25–0.90)	1808	7 (0.39)	0.94 (0.24–1.64)	1.63 (0.64–4.18)
−9	5213	14 (0.27)	0.63 (0.30–0.95)	1926	8 (0.42)	0.99 (0.30–1.68)	1.58 (0.66–3.79)
−8	5498	21 (0.38)	0.90 (0.51–1.28)	2026	16 (0.79)	1.83 (0.92–2.73)	2.03 (1.05–3.92)
−7	5885	17 (0.29)	0.67 (0.35–0.99)	2161	11 (0.51)	1.20 (0.49–1.91)	1.79 (0.83–3.84)
−6	6228	22 (0.35)	0.81 (0.47–1.15)	2277	18 (0.79)	1.89 (1.01–2.76)	2.32 (1.24–4.35)
−5	6546	19 (0.29)	0.67 (0.37–0.97)	2383	17 (0.71)	1.70 (0.89–2.51)	2.54 (1.31–4.92)
−4	6847	31 (0.45)	1.05 (0.68–1.42)	2483	30 (1.21)	2.77 (1.77–3.77)	2.64 (1.59–4.38)
−3	7093	39 (0.55)	1.27 (0.87–1.67)	2565	41 (1.60)	3.64 (2.51–4.78)	2.87 (1.84–4.46)
−2	7225	31 (0.43)	0.98 (0.63–1.32)	2610	39 (1.49)	3.55 (2.42–4.67)	3.63 (2.26–5.85)
−1	7322	34 (0.46)	1.05 (0.70–1.40)	2649	53 (2.00)	4.78 (3.48–6.08)	4.57 (2.96–7.04)
0 (diagnosis year)	7374	41 (0.56)	1.26 (0.87–1.64)	2677	148 (5.53)	13.06 (10.93–15.19)	10.38 (7.33–14.69)
1	7142	22 (0.31)	0.69 (0.40–0.98)	2217	38 (1.71)	4.08 (2.77–5.39)	5.87 (3.47–9.95)
2	6722	40 (0.60)	1.35 (0.93–1.77)	1864	44 (2.36)	5.37 (3.78–6.98)	3.98 (2.58–6.12)
3	6319	39 (0.62)	1.41 (0.97–1.85)	1549	23 (1.48)	3.27 (1.93–4.62)	2.32 (1.38–3.91)
4	5972	25 (0.42)	0.95 (0.58–1.32)	1307	17 (1.30)	2.94 (1.53–4.36)	3.10 (1.67–5.77)
5	5659	36 (0.64)	1.47 (0.99–1.94)	1122	11 (0.98)	2.06 (0.83–3.29)	1.40 (0.71–2.78)
6	5355	22 (0.41)	0.93 (0.54–1.31)	973	7 (0.72)	1.63 (0.42–2.84)	1.76 (0.75–4.14)
7	5108	20 (0.39)	0.89 (0.50–1.28)	852	5 (0.59)	0.71 (0.33–1.58)	0.86 (0.23–2.42)
8	4889	10 (0.20)	0.46 (0.20–0.84)	763	3 (0.39)	0.49 (0.20–1.26)	0.94 (0.21–4.17)
9	4694	11 (0.23)	0.50 (0.21–0.80)	711	2 (0.28)	0.29 (0.14–0.86)	0.51 (0.15–4.64)
10	4541	6 (0.13)	0.31 (0.11–0.56)	652	2 (0.31)	0.26 (0.10–0.87)	0.38 (0.14–6.32)

^a^ Reference group: dementia-free participants.

**Fig. 2:**
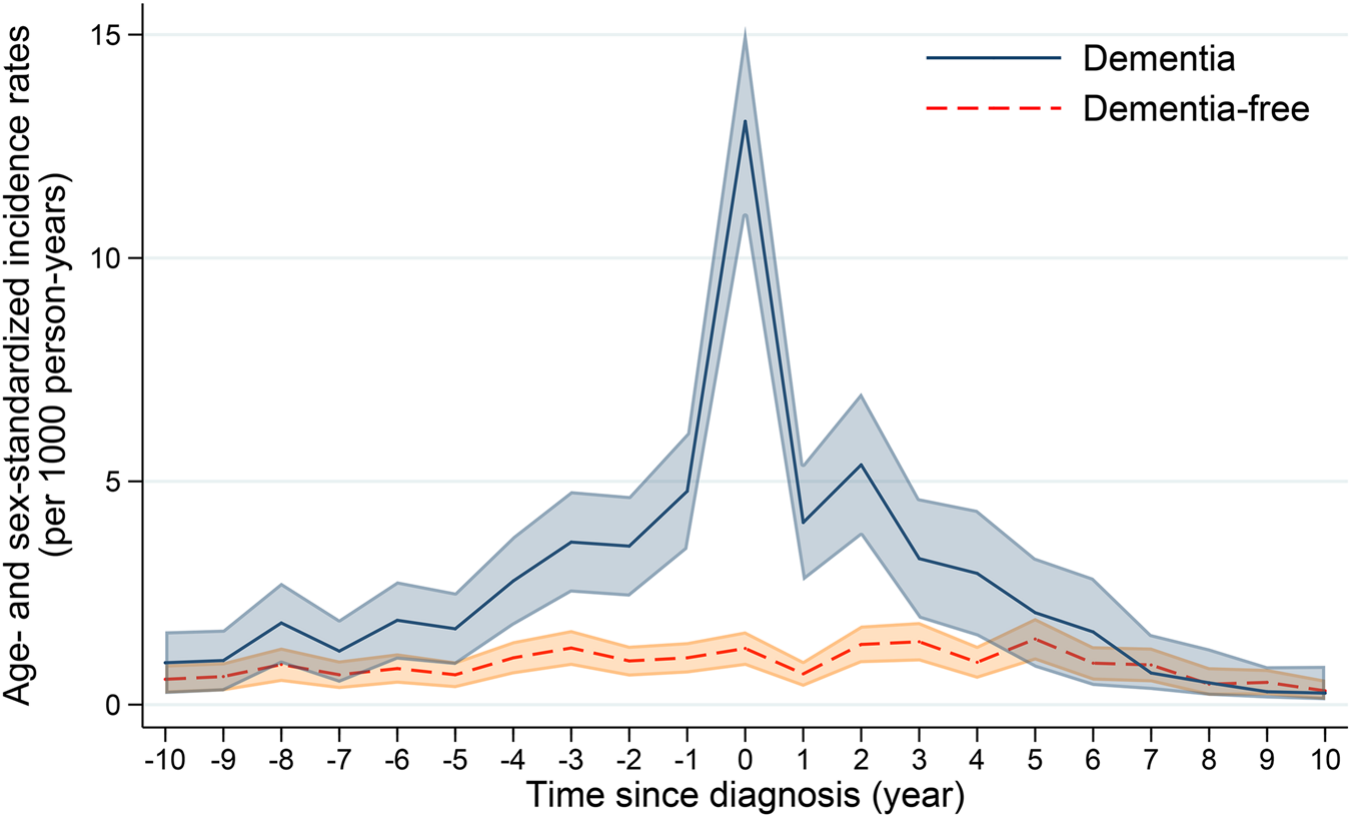
Age- and sex-standardized incidence rates per 1000 person-years and 95% confidence intervals of depression by dementia status (n = 10,051). Shaded areas indicate 95% confidence intervals.

Risk of depression followed a similar pattern. Compared to controls, the risk of depression among participants with dementia increased beginning 6 years before diagnosis (IRR 2.32, 95% CI 1.24–4.35) and reached a peak (IRR 10.38, 95% CI 7.33–14.69) in the year of dementia diagnosis. Depression risk remained elevated among participants with dementia after diagnosis but gradually declined, remaining statistically significant until 4 years post-diagnosis (IRR 3.10, 95% CI 1.67–5.77), after which it became non-significant. (Fig. 2).

### Factors associated with depression before and after dementia diagnosis

**Fig. 3:**
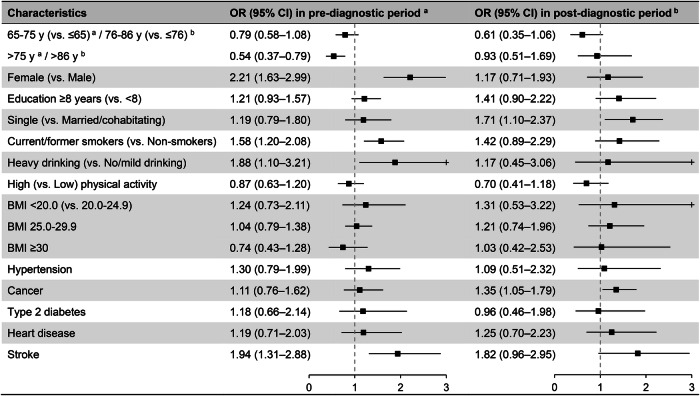
Odds ratios (ORs) and 95% confidence intervals (CIs) for depression-related factors during pre- and post-diagnostic periods in participants with dementia (n = 2677). Models were adjusted for age, sex, education level, marital status, smoking status, alcohol consumption, physical activity level, body mass index (BMI), hypertension, cancer, type 2 diabetes, heart disease, and stroke. + indicates that the 95% CI exceeds the upper limit of the x-axis (3.0). ^a^ Variables were obtained at the time of the Screening Across the Lifespan Twin survey. ^b^ Age, hypertension, cancer, type 2 diabetes, heart disease, and stroke were ascertained 1 year after dementia diagnosis; other variables were obtained at the time of the Screening Across the Lifespan Twin survey.

In the secondary analyses, multi-adjusted GEE models showed that female sex (OR 2.21, 95% CI 1.63–2.99), smoking (OR 1.58, 95% CI 1.20–2.08), heavy drinking (OR 1.88, 95% CI 1.10–3.21), and stroke (OR 1.94, 95% CI 1.31–2.88) were associated with an increased risk of depression during the pre-diagnostic period. Moreover, compared with participants aged <65 years, those aged ≥75 years exhibited lower depression odds (OR 0.54, 95% CI 0.37–0.79). In contrast, in the post-diagnostic period, being single (OR 1.71, 95% CI 1.10–2.37) and having a history of cancer (OR 1.35, 95% CI 1.05–1.79) were associated with a higher risk of depression. No significant associations were observed for education level, BMI, physical activity, hypertension, type 2 diabetes, or heart disease with depression during either the pre-diagnostic or the post-diagnostic period (Fig. 3 and Supplementary Table 2).

## Discussion

In this large-scale study, we found that, compared with dementia-free individuals, those diagnosed with dementia exhibited an increased risk of depression beginning 6 years before dementia diagnosis, peaking in the year of diagnosis, and gradually declining while remaining elevated up to 4 years post-diagnosis. Furthermore, female sex, smoking, heavy drinking, and stroke were associated with higher odds of developing depression during the pre-diagnostic period, whereas being single and having cancer were related to post-diagnostic depression.

During the past decade, the association between depression and dementia has been extensively investigated. However, depression and dementia commonly co-occur later in life and share similar clinical manifestations, complicating the understanding of their temporal dynamics [7, 8]. Assessing depression at multiple time points can help map the occurrence window of depression throughout the dementia timeline. Two previous longitudinal studies characterized the trajectory of depressive symptoms before dementia, suggesting that such symptoms may primarily represent a prodromal feature of dementia [11, 12]. Conversely, other research suggested that earlier-onset depression still confers an increased risk of dementia [29, 30]. On the other hand, depression in dementia patients differs from that in cognitively healthy persons [17]. The progression of dementia and worsening of cognitive function substantially affect mental health, with evidence indicating that nearly all people with dementia would experience one or more episodes of anxiety, depression, or apathy at some point during their illness [16, 31, 32]. Several one-year follow-up studies explored the course of depression following dementia diagnosis, showing that individuals with dementia may be at increased risk of chronic or worsening depression [33, 34].

Building on previous findings, our study took a further step by exploring depression risk in relation to the timing of dementia diagnosis. By simultaneously assessing the trajectory of depression occurrence before, during, and after diagnosis, we found that the likelihood of depression among participants with dementia began to rise 6 years before dementia diagnosis. This suggests that depression may constitute a prodromal phase of dementia. The risk then declined until 4 years after diagnosis. Notably, the bidirectional trajectory of depression in dementia showed that the closer to the time of dementia diagnosis, the greater the likelihood of depression, with peaks occurring immediately before and after diagnosis. This pattern is consistent with previous findings and may reflect overlapping symptomatology or shared pathophysiological mechanisms between depression and the early stages of dementia [29, 35]. However, diagnosing depression among individuals with dementia remains inherently challenging due to shared features, cognitive decline, and communication difficulties. Our findings provide insights into the temporal dynamics of depression in dementia, underscoring the need for stage-specific prevention and management strategies and indicating that these diagnostic challenges merit further study.

Several explanations have been proposed for the relationship between depression and dementia across the disease course. Prior to dementia, depression may contribute to cognitive decline via vascular pathology, hypothalamic-pituitary-adrenal axis dysregulation, neuroinflammation, and reduced neurotrophic support including lower brain-derived neurotrophic factor levels, all of which could lead to neuronal damage and hippocampal atrophy [8, 36–38]. After dementia onset, depressive symptoms may arise as a direct consequence of neurodegenerative changes, such as cortical and limbic neuron loss and disruption of monoaminergic systems, as well as ongoing neuroinflammation and hypothalamic-pituitary-adrenal axis dysregulation [7, 39–41]. Psychosocial factors resulting from dementia, including reduced independence, social isolation, and awareness of cognitive decline, may further exacerbate post-dementia depression [14, 41]. Together, these mechanisms suggest that depression may serve both as a potential risk factor and an early clinical manifestation before dementia, and as a secondary consequence of neuropathology and psychosocial stressors after dementia onset.

In addition, we further examined the factors influencing depression among individuals with dementia. Consistent with a previous study [18], smoking and heavy drinking were associated with an increased risk of depression in the pre-diagnostic phase of dementia. Female sex and stroke were also linked to higher depression risk during this period, potentially related to sex-specific hormonal profiles and stroke-induced neurochemical changes, particularly disturbances in neurotransmitter systems and reduced expression of neurotrophic factors [42]. Interestingly, older age appeared to be related to a lower likelihood of depression compared with younger patients, which may partly reflect survivor bias, as those with both severe depression and dementia at younger ages are less likely to survive into later life. In the post-diagnostic phase, we also found that cancer and being divorced, widowed, or living alone were associated with increased depression risk. A possible explanation is that individuals with dementia may have a reduced capacity to cope with illness-related pain and limited social support, which could intensify the psychological impact of life stressors and prolong depressive symptoms [43]. In line with our findings, a previous report showed that poorer physical health may increase depression levels in older adults with mild cognitive impairment [44]. Collectively, these findings highlight the close interplay among demographic, social, and behavioral factors, physical condition, and mental health in late life, suggesting that targeted interventions addressing lifestyle and comorbid conditions could help mitigate depression risk throughout the course of dementia. Further research is needed to elucidate the risk factors and underlying mechanisms of the onset and progression of psychiatric symptoms in dementia.

Strengths of this study include the use of longitudinal data with repeated yearly assessments of diagnosed depression, which enables us to map its trajectory during the 10-year periods before and after dementia diagnosis. However, several limitations should be acknowledged. First, an important limitation lies in the reliance on depression and dementia diagnoses recorded in the NPR, which predominantly captures more severe cases, such as those receiving hospital or specialized care, and thus misses milder, untreated cases or cases managed in primary care. In addition, dementia is often diagnosed with a delay, meaning that the recorded diagnosis date may not accurately reflect the true onset of the disease [45]. These could result in under-ascertainment of cases and obscure the temporal sequence between depression and dementia. Second, individuals with depression are likely to have more frequent contact with health care services than those without depression, which may increase the likelihood of dementia being detected at an earlier stage. This detection signal bias may have led to an overestimation of the association between depression and dementia. Conversely, the under-ascertainment of both depression and dementia in the registers may have attenuated the association. Taken together, the results of this study may partly reflect the interplay of these opposing biases. Third, information on lifestyle-related factors was collected only once through the SALT survey, making it difficult to capture potential changes in these factors during follow-up. Finally, although we adjusted for a range of potential confounders, some relevant factors, such as social activity engagement and sleep patterns that are associated with depression and dementia [20, 46], were not available in our data and therefore could not be considered.

In conclusion, our study provides evidence that the risk of depression in older adults with dementia begins to increase 6 years prior to dementia diagnosis, peaks in the year of diagnosis, and gradually declines over the following 4 years but remains elevated. Factors including female sex, smoking, heavy drinking, being single, and a history of stroke or cancer were associated with a higher likelihood of depression in this population. Our study highlights the importance of comprehensive depression management across all stages of dementia and reaffirms the need for targeted mental health resources for older adults living with dementia.

## Supplementary information

SUPPLEMENTAL MATERIAL

## Data Availability

The data that support the findings of this study are available from the corresponding author upon reasonable request.
