# Supplementary material for: Trajectory of depression occurrence before, during, and after dementia diagnosis: A population-based study
Source: Transl Psychiatry. 2026 Feb 3;16:124. doi: 10.1038/s41398-026-03817-w (PMC12963397; doi:10.1038/s41398-026-03817-w)
Supplement: Supplementary file 1 — SUPPLEMENTAL MATERIAL [file 41398_2026_3817_MOESM1_ESM.docx]

**Supplementary materials**

This Supplemental material was supporting the methods and results for the manuscript titled Trajectory of depression occurrence before, during, and after dementia diagnosis: A population-based study by Yang et al.

**Supplementary Table 1** International Classification of Disease (ICD) codes of hypertension, type 2 diabetes, heart disease, and stroke

| Diseases | **ICD-7**  (until 1968) | **ICD-8**  (1969-1986) | **ICD-9**  (1987-1997) | **ICD-10**  (1998 onward) |
| --- | --- | --- | --- | --- |
| Hypertension | 444-447 | 400-404 | 401-405 | I10-I15 |
| Type 2 diabetes | 260 | 250 | 250 | E11-E14 |
| Heart disease | 420 | 410-414, 427 | 410-414, 428 | I20-I25, I48-I50 |
| Stroke | 330-334 | 430-438 | 430-437 | I60-I68 |

**Supplementary Table 2** Odds ratios (ORs) and 95% confidence intervals (CIs) for depression-related factors during pre- and post-diagnostic periods in participants with dementia (n=2677)

| Characteristics | Pre-diagnostic period ^a^ | | |  | Post-diagnostic period ^b^ | | |
| --- | --- | --- | --- | --- | --- | --- | --- |
|  | No. of subjects | No. of cases | OR (95% CI) ^c^ |  | No. of subjects | No. of cases | OR (95% CI) ^c^ |
| Age, year |  |  |  |  |  |  |  |
| ≤65 / ≤76 | 621 | 82 | Reference |  | 586 | 23 | Reference |
| 65–75 / 76–86 | 1218 | 126 | 0.79 (0.58–1.08) |  | 1409 | 39 | 0.61 (0.35–1.06) |
| >75 / >86 | 838 | 62 | **0.54 (0.37–0.79)** |  | 682 | 20 | 0.93 (0.51–1.69) |
| Sex |  |  |  |  |  |  |  |
| Male | 1092 | 77 | Reference |  | 1092 | 31 | Reference |
| Female | 1585 | 193 | **2.21 (1.63–2.99)** |  | 1585 | 51 | 1.17 (0.71–1.93) |
| Education level |  |  |  |  |  |  |  |
| <8 years | 1540 | 142 | Reference |  | 1540 | 41 | Reference |
| ≥8 years | 1137 | 128 | 1.21 (0.93–1.57) |  | 1137 | 41 | 1.41 (0.90–2.22) |
| Marital status |  |  |  |  |  |  |  |
| Married/cohabitating | 1672 | 160 | Reference |  | 1672 | 43 | Reference |
| Single | 1005 | 110 | 1.19 (0.79–1.80) |  | 1005 | 39 | **1.71 (1.10–2.37)** |
| Smoking |  |  |  |  |  |  |  |
| Non-smokers | 1619 | 141 | Reference |  | 1619 | 43 | Reference |
| Current/former smokers | 1058 | 129 | **1.58 (1.20–2.08)** |  | 1058 | 39 | 1.42 (0.89–2.29) |
| Drinking |  |  |  |  |  |  |  |
| No/mild drinking | 2570 | 250 | Reference |  | 2570 | 77 | Reference |
| Heavy drinking | 107 | 20 | **1.88 (1.10–3.21)** |  | 107 | 5 | 1.17 (0.45–3.06) |
| Physical activity level |  |  |  |  |  |  |  |
| Low | 492 | 60 | Reference |  | 492 | 21 | Reference |
| High | 2185 | 210 | 0.87 (0.63–1.20) |  | 2185 | 61 | 0.70 (0.41–1.18) |
| BMI, kg/m^2^ |  |  |  |  |  |  |  |
| <20.0 (Underweight) | 147 | 19 | 1.24 (0.73–2.11) |  | 147 | 6 | 1.31 (0.53–3.22) |
| 20.0-24.9 (Normal weight) | 1268 | 128 | Reference |  | 1268 | 35 | Reference |
| 25.0-29.9 (Overweight) | 1047 | 106 | 1.04 (0.79–1.38) |  | 1047 | 35 | 1.21 (0.74–1.96) |
| ≥30 (Obese) | 215 | 17 | 0.74 (0.43–1.28) |  | 215 | 6 | 1.03 (0.42–2.53) |
| Hypertension |  |  |  |  |  |  |  |
| No | 2456 | 246 | Reference |  | 2032 | 60 | Reference |
| Yes | 221 | 24 | 1.30 (0.79–1.99) |  | 645 | 22 | 1.09 (0.51–2.32) |
| Cancer |  |  |  |  |  |  |  |
| No | 2353 | 233 | Reference |  | 2106 | 54 | Reference |
| Yes | 324 | 37 | 1.11 (0.76–1.62) |  | 571 | 28 | **1.35 (1.05–1.79)** |
| Type 2 diabetes |  |  |  |  |  |  |  |
| No | 2405 | 244 | Reference |  | 2287 | 69 | Reference |
| Yes | 272 | 26 | 1.18 (0.66–2.14) |  | 390 | 13 | 0.96 (0.46–1.98) |
| Heart disease |  |  |  |  |  |  |  |
| No | 2230 | 235 | Reference |  | 1927 | 56 | Reference |
| Yes | 447 | 35 | 1.19 (0.71–2.03) |  | 750 | 26 | 1.25 (0.70–2.23) |
| Stroke |  |  |  |  |  |  |  |
| No | 2446 | 233 | Reference |  | 2223 | 66 | Reference |
| Yes | 231 | 37 | **1.94 (1.31–2.88)** |  | 454 | 16 | 1.82 (0.96–2.95) |

^a^ All variables were obtained at the time of the Screening Across the Lifespan Twin survey.

^b^ Age, hypertension, cancer, type 2 diabetes, heart disease, and stroke were collected 1 year after dementia diagnosis; other variables were obtained at the time of the Screening Across the Lifespan Twin survey.

^c^ Models were adjusted for age, sex, education level, marital status, smoking status, alcohol consumption, physical activity level, body mass index, hypertension, cancer, type 2 diabetes, heart disease, and stroke.

BMI = body mass index
